# The Emergent Role of Virtual Reality in the Treatment of Neuropsychiatric Disease

**DOI:** 10.3389/fnins.2017.00491

**Published:** 2017-09-05

**Authors:** Yacine Benyoucef, Pierre Lesport, Amani Chassagneux

**Affiliations:** SPACEMEDEX Valbonne Sophia-Antipolis, France

**Keywords:** augmented reality, cybertherapy, neuropsychiatric disorder, pain, rehabilitation medicine, virtual reality

## Background

The development of outstanding computer science technologies such as virtual reality (VR) and augmented reality (AR) has provided new avenues in order to challenge the functional connectivity disruption seen in neurodegenerative diseases.

Virtually simulating the outside world with high realism, close-to-total immersion and easiness of interaction presents an interesting way to decipher new therapeutic perspectives as a non-invasive and non-pharmacological neuromodulation tool.

As of recently, these cutting-edge techniques already appear to be used in the treatment of some neuropsychiatric disorders, cognitive and behavioral impairments since the patient is placed in an environment virtually yet specifically adapted to his needs. Specific use of VR and AR becomes interesting for both social and functional rehabilitation whether it originates from traumatic situations and injuries or from pathologies known to show from neuromuscular disturbances to decay. Virtual reality is already used in research in combination to rehabilitation adapted treadmills in order to help treat children with cerebral palsy (coordination impairment, stiff and weak muscles, tremors, etc.) that appear in early childhood (Sloot et al., 2015; Cho et al., 2016). However, there is still some work to be done to improve the actual research setups and the reproducibility of results.

Therefore, we will review here the innovative and emergent role of cybertherapies and focus on the possibilities to extend its use as far as rehabilitation centers to everyday well-being.

## Neuropsychiatric perspectives

Nowadays, virtual simulations of our world are created with high realism coupled to immersion techniques. This appears to be a useful tool in the treatment of neuropsychiatric diseases and behavioral disorders which might concern almost 450 million of patients worldwide. Statistically, one out of four people has already or will develop a mental disorder during their lifetime according to the World Health Organization (WHO, 2001). And because virtual reality allows for a wide range of applications, this cutting-edge technology could find its place as a neuromodulator to target a panel of states from wellbeing to mental diseases and pain management.

But, due to its innovative nature, there is a strong need to enhance its development and to standardize applications. So far, specific adaptative environments and serious games are selected as a means to a safe and regular use in clinical medicine. It constitutes in itself a brand new public health approach to help treating brain disorders: troubled thinking and perception disorders, cognitive impairment, disruption of emotions, or behavioral disorders are some of the areas targeted by this cutting-edge treatment using serious games: the patient is in a specific virtual environment and evolves as he would in his real life. The objective is to help him face his fears or behavioral challenge in order to improve his day-to-day life (Malbos et al., 2013; Sarver et al., 2014; Fleming et al., 2017).

Traditional exposure methods were found in protocols such as gradual *in vivo* exposure, role-playing games and sometimes group therapy but all eventually led to a similar limitation due to systematic desensitization (Cottraux, 1994). Success is often reached with difficulties because of limitations like the lack of imagination for the patient, usually coupled to a lack of intimacy and aversion to participate or the cost of such a therapy. All of which don't appear with the virtual reality, since VR allows for a private use at a lower cost: it has to be used as an additional technique to overcome limitations of traditional psychotherapies. Through VR the idea is to dive deep into an anxiogene and interactive environment with a controlled, safe and ludic protocol. A strong planning is mandatory, as every patient needs a design specifically matched to his pathology. One of the main challenges is to stick to the reality as accurately as possible in order to solve or at least lessen the psychopathological issue caused by the dismemberment of the conscience, which appears like a conflict between stimuli and cognition, between what we sense and what we live. This paradigm could be resolved by a full presence feeling: the more we are in, the less we are in conflict, and the more the treatment is effective.

That presence feeling could be brought by the AR technique, where the system allows inserting virtual contents in the real world as a real time perceived ultra-reality. Virtual elements are superimposed over the real image of the environment, bringing additional information and augmenting our perception. That technology is already powerful in medical training (Cutolo et al., 2017) and as a navigational tool in surgery (Cabrilo et al., 2014), but appears to be useful in the treatment of specific phobia, specifically on the phobia of small animals (Chicchi Giglioli et al., 2015), even if there is still a strong protocols development and clinical tests to do to extend its use not only in phobia but also in other pathologies.

## Rehabilitation medicine

Associated with specific exercises, VR assisted rehabilitation becomes interesting for both social and functional rehabilitation (Laver et al., 2015; Dockx et al., 2016; White and Moussavi, 2016; Fernandez Montenegro and Argyriou, 2017). The overall system using a treadmill or an exoskeleton plus a chosen specific virtual environment might have an important impact for post-traumatic injuries or diseases with neuromuscular disturbances and degeneration (Villiger et al., 2013; Pietrzak et al., 2014). Using the specified setup, the patient will be able to walk and move, repeating the exercise and gradually increasing the intensity. Such systems could bring an innovative solution through the use of virtual reality, where objectives, motivation, and targeted exercises could be mixed in a ludic protocol. Indeed, VR assisted rehabilitation allows the patient to work on locomotion disorders, postural rehabilitation, and equilibrium as well as spatial navigation or any other disorders (Chiarovano et al., 2017; Cogné et al., 2017). Patients with memory impairment such as witnessed in Alzheimer's disease could benefit from experiencing a virtual walk outside without putting them at risk of getting lost and VR could help creating familiar environments with for example the incorporation of musicotherapy and family pictures. With an adapted setup, both young patients and senior patients could see their rehabilitation sessions benefiting from a new vision and therefore see a new capacity added to the means accessible to them in order to get better. By targeting specific brain areas and specific training, VR could be used as a cortical reorganization inducer, and trigger new healing neurological and critical pathways in mental health disorders or trauma induced stress and anxiety such as Post-Traumatic Stress Disorder (Maples-Keller et al., 2017; Tielman et al., 2017), to adaptive rehabilitation following stroke induced neurological impairments (Xiao et al., 2017; Yasuda et al., 2017).

The next step would be to integrate haptic control and grabbing of virtual elements for the patient to be immersed in an even more lifelike simulated reality in order to achieve greater stimulation and to obtain enhanced results aiming at improving living conditions for all patients suffering from myopathies.

As of late, software improvements and reduction in the costs of virtual gear transformed VR into a practical tool for immersive multisensory experiences in 3D that can distract patients from their chronic pains. Virtual reality is a promising intervention with several potential applications in the inpatient medical setting. Studies to date demonstrate some efficacy, but there is a need for larger, well-controlled studies to show clinical and cost-effectiveness (Dascal et al., 2017). Several studies aiming to assess the effectiveness of VR in hospitalized patients with chronic pains have been published. The use of VR as a distraction in these patients was effective to a point in reducing pain compared to a control distraction condition (Jin et al., 2016; Jones et al., 2016; Garrett et al., 2017; McSherry et al., 2017; Tashjian et al., 2017), showing that it can be used as a bonus therapy tool for pain management. But these findings are coming in contradiction to other studies that point out that the use of VR didn't change pain thresholds in patients (Smith et al., 2017). VR and AR were also studied in the context of Phantom Limb Pain (PLP). The PLP patients present a perception of discomfort consecutive to amputation of their limb. In such cases, mirror therapy is used and VR and AR therapy can provide an increased level of immersion with customizable environments for the amputee to alleviate the discomfort. They can also use myoelectric sensors to manipulate artificial limbs to perform several tasks, thus limitating their handicap (Ortiz-Catalan et al., 2014; Dunn et al., 2017).

The pain management of patients in hospital could nonetheless benefit from VR as adjunctive therapy if it is used in the right emotional and social context. For example, the qualitative effects of immersive virtual reality therapy during painful procedures are possible in the operating theater environment and could contribute in postoperative satisfaction (Mosso-Vázquez et al., 2014; Chan and Scharf, 2017). More insights will be obtained by future research with more immersive VR technology to explore the potential link between stimuli and the context that changes the value of these stimuli. VR was also successful as adjunct therapy for acute pain management but most of the studies reported very important individualized responses in patients (Garrett et al., 2017).

## Conclusion

As such, the efficiency of VR and AR, while attractive due to the increasing levels of immersion, customizable environments, and decreasing costs, is yet to be fully proven and continues to need further research with higher quality studies to fully explore its benefits and democratize its use.

## Author contributions

All authors worked from the conception of this article to the final approval, and share the same opinion.

### Conflict of interest statement

Spacemedex is a start-up in the field of aerospace medicine, biotechnologies and medical devices. The corresponding author is the founder and chief executive officer. All other authors have worked as consultants in neuroscience and collaborate to the successful development of our research policies.
